# The clinical diagnosis of Parkinson's disease

**DOI:** 10.1055/s-0043-1777775

**Published:** 2024-02-07

**Authors:** Renato P. Munhoz, Vitor Tumas, José Luiz Pedroso, Laura Silveira-Moriyama

**Affiliations:** 1 University Health Network, Toronto Western Hospital, Morton and Gloria Shulman Movement Disorders Centre and the Edmond J. Safra Program in Parkinson's Disease, Toronto, ON, Canada.; 2 Krembil Research Institute, Toronto, ON, M5T 2S8, Canada.; 3 Universidade de São Paulo, Faculdade de Medicina de Ribeirão Preto, Departamento de Neurociências e Ciências do Comportamento, Ribeirão Preto SP, Brazil.; 4 Universidade Federal de São Paulo, Departamento de Neurologia, São Paulo SP, Brazil.; 5 Universidade Estadual de Campinas, Campinas SP, Brazil.; 6 UCL Queen Square Institute of Neurology, London, United Kingdom.

**Keywords:** Parkinson Disease, Supranuclear Palsy, Progressive, Multiple System Atrophy, Atherosclerotic Parkinsonism, Secondary Parkinsonism, Doença de Parkinson, Paralisia Supranuclear Progressiva, Atrofia de Múltiplos Sistemas, Parkinsonismo Secundário, Parkinsonismo Aterosclerótico

## Abstract

After more than 200 years since its initial description, the clinical diagnosis of Parkinson's disease (PD) remains an often-challenging endeavor, with broad implications that are fundamental for clinical management. Despite major developments in understanding it's pathogenesis, pathological landmarks, non-motor features and potential paraclinical clues, the most accepted diagnostic criteria remain solidly based on a combination of clinical signs. Here, we review this process, discussing its history, clinical criteria, differential diagnoses, ancillary diagnostic testing, and the role of non-motor and pre-motor signs and symptoms.

## Resumo

Passados mais de 200 anos desde a sua descrição inicial, o diagnóstico clínico da doença de Parkinson (DP) continua a ser um processo muitas vezes desafiante, com amplas implicações que são fundamentais para o manejo clínico. Apesar dos grandes desenvolvimentos na compreensão da sua patogénese, marcadores patológicos, características não motoras e potenciais pistas paraclínicas, os critérios diagnósticos mais aceitos permanecem solidamente baseados numa combinação de sinais clínicos motores. Aqui, revisamos esse processo, discutindo sua história, critérios clínicos, diagnósticos diferenciais, testes diagnósticos complementares e o papel dos sinais e sintomas não motores e pré-motores.

## INTRODUCTION

In 1817 James Parkinson described the clinical characteristics of 6 patients who had a neurological syndrome that had not yet been well characterized, which he called ” paralysis agitans ” or “shaking palsy” 1 . In his observations, Parkinson captured main clinical features such as the insidious onset with a progressive disabling course, the presence of resting tremors with asymmetrical body involvement, postural changes with flexion of the trunk, neck, and limbs, abnormal gait with festination, presence of dysarthria, dysphagia, and drooling. He also described the presence of constipation and cognitive preservation.

The description of this new syndrome was slowly incorporated into the medical literature of that period, and at the end of the 19th century, two authors made important contributions. 2 3 4 Trousseau described the presence of muscular rigidity and the progressive slowing of repetitive movements, also noting that patients developed cognitive decline as the condition progressed. Charcot defined bradykinesia as one of the most important manifestations of the disease and the main source of motor disability. He suggested the eponym Parkinson's disease (PD) celebrating the original descriptor. Charcot also noted that there were clinical variants of this syndrome with atypical presentations without tremor, with extension rigidity, with hemiplegia, and “astonished face”.

At the beginning of the 20th century, between 1917 and 1926, the encephalitis lethargica pandemic left post-encephalitic parkinsonism as a sequelae, which was the first recognized secondary cause of parkinsonism. At that time, authors like Critchley tried to characterize various Parkinson-like syndromes, such as “atherosclerotic parkinsonism”, already recognizing the heterogeneity of the syndrome and its probable etiologies. 4 Besides, studies by many authors including Lewy, Tretiakoff, Marinesco, Foix, and Nicolesco made it possible to determine that alterations in the substantia nigra compacta and the presence of Lewy bodies (LBs) were the essential pathological substrate of PD.

In 1967, Hoehn and Yahr wrote their seminal study on parkinsonism in the pre-levodopa era. 5 They described the clinical characteristics of 802 patients with “ *all of the accepted cardinal signs of parkinsonism: rest tremor, plastic rigidity, paucity or delayed initiation of movement, slowness, and impaired postural and righting reflexes* ”. PD was defined as the primary or “idiopathic” form of the disease. The suspicion of an underlying process that could be considered etiologic in inducing the clinical signs, or the presence of associated or atypical neurologic abnormalities, excluded a given case from this idiopathic diagnostic category. The authors defined secondary parkinsonism when the syndrome was linked to a potential etiologic agent and/or there were signs suggesting that parkinsonism was part of a pathologically broader disease affecting systems not typically involved in the archetypal syndrome. These secondary cases were classified as postencephalitic parkinsonism or “others”. Finally, a certain proportion of cases were classified as having indeterminate parkinsonism, as they were deemed impossible to determine whether the clinical signs were primary or secondary. As such, the possibility of different causes for parkinsonism was already well recognized and the differential diagnosis was based on the clinician's impressions.

At that time, it was already not infrequently acknowledged that the diagnosis of PD could be challenging and mistaken for aging-related gait alterations, mobility limitations secondary to joint abnormalities, and especially for cases of essential tremor and neuroleptic-induced parkinsonism. Concurrently, the degenerative diseases that would later be considered the main causes of atypical parkinsonism are very well described [multiple system atrophy (MSA), progressive supranuclear palsy (PSP), and corticobasal degeneration (CBD)]. 6 7 8 Also in the 1960s, studies showed that LBs, characteristic of PD, could be found in the brains of elderly people who died asymptomatic or who had discreet and dubious signs of parkinsonism. 9 10 These observations led to the hypothesis that there was a prodromal phase preceding the appearance of the typical signs of PD. It would later become clear that there must be significant neuronal loss in the substantia nigra compacta and severe striatal depletion of dopamine for the signs of parkinsonism to surface. 11

In the 1970s, the therapeutic revolution in this field began with the use of levodopa. It soon became clear that some patients diagnosed with PD did not respond to treatment, and that it was common for many to develop levodopa-induced dyskinesias. 12 In the 1980s, the term Parkinson-plus began to be used to designate cases of parkinsonism with a supposed neurodegenerative etiology that “mimics PD” added by additional or atypical clinical features such as cerebellar or pyramidal signs. 13 At this time, clinical-pathological studies carried out in the UK by Gibb and Lees outlined for the first time the clinical characteristics that best distinguished PD from other pathologic conditions that also cause parkinsonism. 14 15 These studies also gave rise to the first well-defined diagnostic criteria for PD, discussed next.

## DIAGNOSTIC CRITERIA

The diagnosis of PD has evolved considerably over the last decades. One of the main advances was the furthering of the understanding of differential diagnoses, with the description of MSA 16 and PSP 8 in the 1960s. The next decades of literature were marked by better delineation of the clinical features of PD which could lead to higher diagnostic accuracy and the seminal paper from Gibb and Lees 10 , which is cited as the original source of the Queen Square Brain Bank (QSBB) Criteria for the diagnosis of PD. The manuscript looked at the age-specific prevalence of LBs in the brains of 273 individuals who did not suffer from PD, showing a growing proportion of brains positive for the inclusion from 3.8% to 12.8% between the sixth and ninth decades. The “UK Parkinson's Disease Society Brain Bank clinical diagnostic criteria” (later renamed QSBB Criteria) is mentioned in the introduction and described in detail in a table describing the diagnostic process for PD. Step 1 consists of identification of Parkinsonian syndrome. Bradykinesia is an obligatory criterion for the syndrome, and it is defined as “slowness of initiation of voluntary movement, with a progressive reduction in speed and amplitude of repetitive actions”. This definition of bradykinesia was a powerful ally in differentiating bradykinesia from slowness in other conditions such as dystonia, altered mental states, depression, etc. Step 2 was the exclusion of findings that could point to alternative diagnoses including findings in history (stepwise decline, repeated head trauma, encephalitis, or treatment with dopamine receptor blocking agents at onset), neurological examination (oculogyric crises, supranuclear gaze palsy, cerebellar signs, Babinski signs) or disease course (early severe dysautonomia or dementia, unilateral disease after 3 years). And finally, Step 3 was the presence of supportive criteria. The QSBB Criteria proposes the following features as supportive criteria: occurrence of rest tremor, unilateral onset with ongoing asymmetry, evidence of progression, consistent levodopa response (>70%), levodopa-induced chorea, levodopa response for more than 5 years, long clinical course (>10 years). 10

The QSBB Criteria became the most widely used criteria for the diagnosis of PD in the subsequent years, and by the 1990s the clinical accuracy of the diagnosis of PD had significantly increased to up to 90% in the hands of specialists. 17 Slowly small changes were made to the criteria, including ignoring the exclusion of hereditary cases, since it became clear that certain genetic disorders including mutations in alpha-synuclein 18 and in LRRK2 19 20 could cause a form of PD that could be clinically identical to idiopathic PD both from the clinical point of view and the neuropathological as well since both presented with LBs and Lewy neurites with alpha-synuclein accumulation. 21 The advent of ancillary tests which could show abnormalities in PD cases started to be incorporated into clinical practice, mainly the use of olfactory tests, 22 cardiac imaging using MIBG, 23 and functional imaging of the dopaminergic pathways. 24 With the growing interest in scientific studies in PD it also became important to include different levels of certainty on the diagnosis, enabling better diagnostic certainty using criteria with high specificity for recruitment in clinical studies and empirical management in daily practice. In 2015 The International Parkinson and Movement Disorder Society (MDS) created a new set of criteria, to include these concepts and further improve the accuracy of the diagnosis. 25 The QSBB and the new MDS criteria are compared in Table 1 . The central part of the diagnosis did not change significantly, but two different diagnostic categories were created: Clinically Established PD and Clinically Probable PD. The first level uses criteria for higher specificity, while the second tries to achieve a balance between sensitivity and specificity, to include a larger number of PD cases (that would not make the cut for clinically established) without including too many false positives. In addition, the MDS also created derivative criteria to be applied to early disease when diagnosis is more challenging, mainly for the purpose of clinical trials. 26

**Table 1:** Comparison of the QSBB and the new MDS criteria.

Criteria	Queen Square Brain Bank Criteria (Gibb & Lees, 1988) 10	MDS criteria for Parkinson's disease (Postuma et al., 2015) 25
Chore findings	STEP 1: identification of Parkinsonian syndrome. Defined as bradykinesia and at least one of the following: • Muscular rigidity. • 4-6 Hz rest tremor. • Postural instability not caused by primary visual, vestibular, cerebellar, or proprioceptive dysfunction.	The first essential criterion is parkinsonism, which is defined as bradykinesia, in combination with at least 1 of rest tremor or rigidity.
Negative features used as	Step 2: Exclusion Criteria	Absolute exclusion criteria or Red flags (when combined with supportive criteria do not exclude PD)
Positive features used as	Step 3: Supportive Criteria	Supportive Criteria
Ancillary tests	Imaging used to exclude differential diagnosis (Step 2)	Olfactory loss or cardiac sympathetic denervation on MIBG scintigraphy are supportive criteria Normal functional neuroimaging of the presynaptic dopaminergic system is an exclusion criteria
Certainty levels	Definite PD (three or more positive supportive findings)	Clinically Established PD: 1. Absence of absolute exclusion criteria 2. At least two supportive criteria, and 3. No red flags Clinically Probable PD: 1. Absence of absolute exclusion criteria 2. Presence of red flags counterbalanced by supportive criteria - 1 red flag requires at least 1 supportive criterion - 2 red flags require at least 2 supportive criteria - no more than 2 red flags are allowed for this category

## DIFFERENTIAL DIAGNOSIS OF PARKINSON'S DISEASE

The hypernym term parkinsonism refers to the concomitant finding of two or more out of four signs: bradykinesia, resting tremor, rigidity, and postural instability, 27 invariably including PD as the most common etiologic diagnosis. The term, however, encompasses expanding and variable subsets of disorders that conform to this criterion, including secondary forms [e.g., infectious, drug-induced (DIP), vascular parkinsonism(VP)], sporadic [“atypical parkinsonism” e.g., MSA, PSP, CBD, Lewy body dementia (LBD), etc.], and heredodegenerative disorders [e.g., Wilson's disease (WD), Huntington's disease (HD), spinocerebellar ataxias (SCA)]. 28 Physiopathologically, these disorders have at least one common feature: disruption of the nigrostriatal pathway, induced by chemical, structural, or, more often, degenerative abnormalities leading to flawed control of voluntary movements. 17 Suboptimal diagnostic accuracy, unfortunately, is not rare as noted in two important clinicopathological studies that found a matching ante- and post-mortem diagnosis in 76% of PD cases, ranging from 41 to 88% in cases with pathologic diagnosis of MSA and PSP. 29 30 31 32 These findings have important implications both on clinical and research grounds as a wrong final diagnosis may distort the results of epidemiological, therapeutic, and genetic studies, and misguide management and prognostic aspects related to each of these syndromes. Finally, although most of the differential diagnoses of PD have their own established diagnostic criteria, the phenotypes often overlap and they do not have objective pathognomonic clinical or paraclinical findings. 29

Table 2 describes the main different Parkinsonian syndromes, their features, and clues for diagnosis.

**Table 2:** Clinical features of the most common differential diagnoses of the syndrome. 27 28 29 30 31 32

	PD	DIP	VP	PSP	MSA-P	LBD	CBD
**Mean age of onset (SD)**	59.4 (11.5)	60.6 (13.4)	70.6 (6.4)	66.9 (7.6)	55.5 (6.5)	67.8 (9.2)	63 (7.7)
**Tremor**	Pure rest (30%), pure action (20%), mixed (20%)	Pure rest (35%), pure action (10%), mixed (30%)	Pure rest (4%), pure action (10%), mixed (2%)	Pure rest (10%) ^a^ , pure action (20%), mixed (20%)	Rest (5%), Action (80%) ^f^ , mixed (10%) ^f^	Pure rest (3%), pure action (7%), mixed (24%)	Rest (2%), Action (10%) ^g^ , mixed (55%) ^g^
**Postural instability**	Common but late feature	Rare	Prominent / early or presenting sign	Prominent / early or presenting sign	Prominent / early	Prominent / early	Prominent / early
**Asymmetry**	+++	0	+	0 ^a^	+	0	+++
**Survival – Mean (SD)**	Variable ^b^	N/A	8 (4.1)	8.6 (5.7)	7.5 (4)	4.1 (4.1)	8 (0.7)
**Levodopa response**	Marked / sustained	None to moderate ^c^	None to moderate ^c^	Mild to moderate ^d^	Mild to moderate ^d^	Mild to moderate ^d^	Mild ^d^
** LID ^e^**	++ + +	0	+	+ ^a^	++	+	+
**Dementia**	Common in advanced stages	0	Very common, presenting as VD	Very common, early, fast decline	Less common than PD	Part of diagnostic criteria; may fluctuate	Common, may be early, fast decline
**RBD**	Very common	0	0	Unusual	Very common	Very common	0
**Additional clinical features**	Slower progression compared to other degenerative forms.	Onset during treatment with offending drug; improvement / resolution after withdrawal.	Pyramidal and pseudobulbar signs; lower body predominant.	Supranuclear gaze palsy; disproportional axial (nuchal) rigidity; photophobia / blepharospasm;	Profound early dysautonomia; anterocollis; pseudobulbar affect; pyramidal signs.	Early well-formed visual hallucinations; neuroleptic sensitivity; dysautonomia.	Limb dystonia; apraxia; cortical sensory loss; alien limb phenomena.
**Brain MRI findings**	No specific findings on standard imaging.	No change	Periventricular white matter lesions, lacunar infarcts in BG, ventricular dilation.	Predominant midbrain atrophy; superior cerebellar peduncle atrophy.	Putaminal atrophy; OPCA and “hot cross bum sign” in advanced stages.	Global atrophy.	Asymmetric fronto-parietal atrophy.

Abbreviations: PD, Parkinson's disease; DIP, drug-induced parkinsonism; VP, vascular parkinsonism; PSP, progressive supranuclear palsy; MSA-P, Parkinsonian form of multiple system atrophy; LBD, Lewy body dementia; CBD, corticobasal degenerartion; SD, standard deviation; LID, levodopa-induced dyskinesia; RBD, REM-sleep behavior disorder; MRI, magnetic resonance imaging; BG, basal ganglia; OPCA, olivo-ponto-cerebellar atrophy.

Notes: a) PSP-P variant presents with asymmetric features, rest tremor, levodopa response and LID; b) widely dependent on age of onset, ranging from 38 (5) years for early onset (25-39 years old) to 5 (4) for late onset (≥ 65 years old); c) may be sustained in responders; d) typically in early stages, not sustained; e) in levodopa responders under long term treatment; f) jerky postural tremor / polyminimyoclonus; g) jerky action tremor / myoclonus.

## PRODROMAL PARKINSON'S DISEASE

At the moment, criteria for diagnosis of PD are based on the finding of a combination of motor symptoms and signs as previously stated in this review. 27 However, multiple lines of evidence unequivocally show that by the time when these features surface to clinical detection, pathological and neurochemical hallmarks of the disease are already established and have been already in progress for a considerable amount of time. 33 As such, the quest for a “pre-motor syndrome” delineating potential non-motor features that, alone or in combination, could have enough specificity to suggest the eventual PD diagnosis is of importance for multiple reasons, including the opportunity to contemplate interventions aimed at slowing or stopping disease progression at the earliest pathological stages, even before nigro-striatal degenerative neuronal damage is severe enough to set off early motor dysfunction. 34 35 The groundwork for this endeavor, based on their clinical aspects rather than functional or pathological facets and implications, is discussed below.

## OLFACTORY DEFICITS AND HYPOSMIA

The investigation of olfactory deficits in PD dates back almost half a century, 5 with early observations highlighting its emergence as a potential pre-motor sign. 22 Over the years, research has consistently demonstrated abnormalities in odor discrimination, detection threshold, and identification in PD patients, irrespective of various clinical parameters. 36 37 Since then, the literature explored this topic trying to elucidate the multifaceted nature of olfactory dysfunction in PD, exploring its association with dopaminergic and cholinergic mechanisms, the presence of LBs, and its implications for early diagnosis. Current available studies span for decades, encompassing diverse patient populations in terms of age of onset, disease duration and severity, motor laterality, phenotype, treatment status, and cognitive impairment. These investigations employed methods ranging from clinical assessments of olfactory sensitivity to post-mortem examinations, aiming to unravel the intricate relationship between olfactory dysfunction and PD. Contrary to initial expectations, olfactory dysfunction in PD does not exhibit a direct correlation with dopaminergic dysfunction or the motor signs characteristic of the disease. 38 Instead, evidence suggests that cholinergic deficits, particularly in the limbic cortex, play a more substantial role in determining olfactory deficits in PD than nigrostriatal dopaminergic denervation. The presence of LB in the olfactory bulb emerges as a consistent pathological marker in symptomatic PD patients, occurring in virtually all cases, building upon the hypothesis proposed by Braak et al., 33 which posits that the degenerative process in PD initiates in the olfactory bulb and anterior olfactory nuclei, leading to olfactory sensitivity loss in 70%-90% of PD patients, including those who are treatment-naïve and newly diagnosed. 36 This supports the notion that hyposmia serves as a pre-motor sign, with LB consistently found in the substantia nigra pars compacta (SNc) alongside these pathological markers in olfactory structures. However, the temporal relationship between the onset of hyposmia and the manifestation of motor signs remains uncertain, with a potential lag of several years. 39

In summary, olfactory dysfunction in PD presents a complex interplay of neurobiological factors involving dopaminergic and cholinergic systems, as well as the presence of LB in specific areas. Understanding the nuances of olfactory deficits not only contributes to the elucidation of PD's pathophysiology but also offers valuable insights for early diagnosis. Moreover, the distinct patterns of hyposmia observed in PD, MSA, PSP, and CBD underscores its potential utility as a diagnostic marker in differentiating Parkinsonian syndromes. Further research is warranted to unravel the temporal dynamics of olfactory dysfunction and its role in the prodromal phase of PD. 39

### REM sleep behavior disorder

Rapid eye movement (REM) Sleep Behavior Disorder (RBD) is a distinctive parasomnia characterized by the loss of normal muscle atonia during the REM sleep phase. This phenomenon results in the enactment of dream content, often involving vocalizations and complex movements. In the context of PD, similar to hyposmia, RBD has emerged as a potential pre-motor sign, providing valuable insights into the neurodegenerative process. 40

During REM sleep, intricate patterns of neuronal activation and neurotransmitter release occur in the brain stem, leading to motor inhibition and muscle atonia. RBD disrupts this normal physiological process, causing individuals to act out their dreams, sometimes resulting in sleep disturbances and injuries. This abnormality is particularly prevalent in PD patients, suggesting a unique distribution of the degenerative process in these individuals. 40 The gold standard for diagnosing RBD involves polysomnography, revealing excessive muscle activity, and increased submental electromyography (EMG) density during REM sleep. 41 Clinically, historical data, ranging from formal criteria outlined in the Manual of Disorders of Sleep of the American Academy of Sleep Medicine 42 to a simple yes/no questionnaire, can aid in diagnosis. 43 44

PD patients with RBD exhibit distinct clinical features, including worse postural instability and gait, suboptimal motor response to levodopa, orthostatic hypotension, visual color perception deficit, visual hallucinations, and an increased risk of developing dementia. 41 RBD often precedes the onset of motor symptoms in PD, with a mean interval of 1 to 12 years. 41 Notably, individuals with apparently idiopathic RBD face a greater than 50% chance of developing neurodegenerative diseases after 12 years of follow-up, most commonly PD, followed by LBD, Alzheimer's disease, and MSA. 45 While RBD is frequently associated with synucleinopathies, particularly PD, LBD, and MSA, its occurrence in atypical Parkinsonian syndromes such as PSP suggests a complex relationship between the disorder and the topographic progression of the degenerative process. 46 Understanding the intricacies of RBD in the context of PD contributes valuable insights into both diagnostic approaches and the underlying neurobiology of these conditions. Further research is warranted to elucidate the specific molecular and topographic factors influencing the manifestation of RBD across diverse neurodegenerative diseases.

### Mood disorders

Depression and anxiety are prevalent in PD, affecting more than a quarter of newly diagnosed cases. Studies indicate that individuals with depression are 2.2 to 3.2 times more likely to develop PD compared to healthy controls. 47 While the correlation is less conclusive than for other symptoms, such as hyposmia and RBD, depressive symptoms may precede motor signs, peaking around 3-6 years before a PD diagnosis. 35 A study involving 1,358 patients with depression found a 13.3 times higher chance of developing PD compared to controls without depression. 48 Another study reported a 2.95 times higher likelihood of PD occurrence in individuals with depression. In summary, current evidence considers depression as a risk factor for PD, though not necessarily a pre-motor symptom. 49

### Constipation

Constipation is a common pre-motor symptom in PD, often present at diagnosis and extending over a variable period, up to 24 years before the onset of parkinsonism. 35 A longitudinal study with 6,790 males revealed a 2.7 times higher risk of PD in individuals with constipation. The time interval between constipation detection and PD diagnosis averaged 12 years. 50 Pathologically, alpha-synuclein aggregates in the peripheral autonomic system contribute to this relationship, affecting abdominal-pelvic, cardiac, and myenteric plexus. 51 Constipation may reflect both peripheral and central mechanisms, indicating pelvic floor dysfunction. Some individuals with constipation also exhibit LB in the central nervous system, as well as pre-motor signs like RBD or striatal abnormalities. 35 51

### Weight loss

PD patients often have a lower body mass index (BMI) compared to healthy controls, attributed to factors like dyskinesias, changes in eating habits, medication effects, and prolonged meal ingestion leading to lower energy intake. 52 Studies have explored physiological changes, such as altered levels of leptin, insulin-like growth factor type 1 (IGF-1), and thyroid-stimulating hormone in PD patients with weight loss. 53 Weight loss in PD is multifactorial and may occur before or throughout the disease stages. A prospective study showed that BMI remained stable in most patients until a variable period before motor symptoms appeared, ranging from a few months to four years. 54

### Effect of pre-motor features on PD prediction

The effect of single and concomitant pre-motor features on prediction of PD: Although there is enough evidence to support the pre-motor nature of these signs and symptoms, their sensitivity and specificity are not high enough to call them generically “predictors” (RBD may be an exception to this statement though). Based on the prevalence of the manifestations in early disease, the maximal sensitivity favors hyposmia, while specificity is best for RBD. However, the combination of the two indicates a more than four-fold increase in the probability of PD on longer follow-up compared to presenting one of these features alone. 55

## ANCILLARY INVESTIGATION FOR THE DIAGNOSIS OF PARKINSON'S DISEASE

As aforementioned, the diagnosis of PD is essentially performed based on clinical observation. However, several additional tests play an important role in its differential diagnosis with other movement disorders, such as essential tremor (ET) and atypical parkinsonism. 27 56 Also, genetic testing may add important tools for counseling regarding inheritance, prognosis, and even treatment choices.

### Neuroimaging in Parkinson's disease

Routine brain magnetic resonance imaging (MRI) is usually unremarkable in patients with PD. The value of brain MRI in this context lies in ruling out structural abnormalities, secondary causes of parkinsonism (i.e., VP and normal pressure hydrocephalus) and identifying changes often seen in atypical parkinsonism, such as MSA and PSP. 57

**Figure 1:**
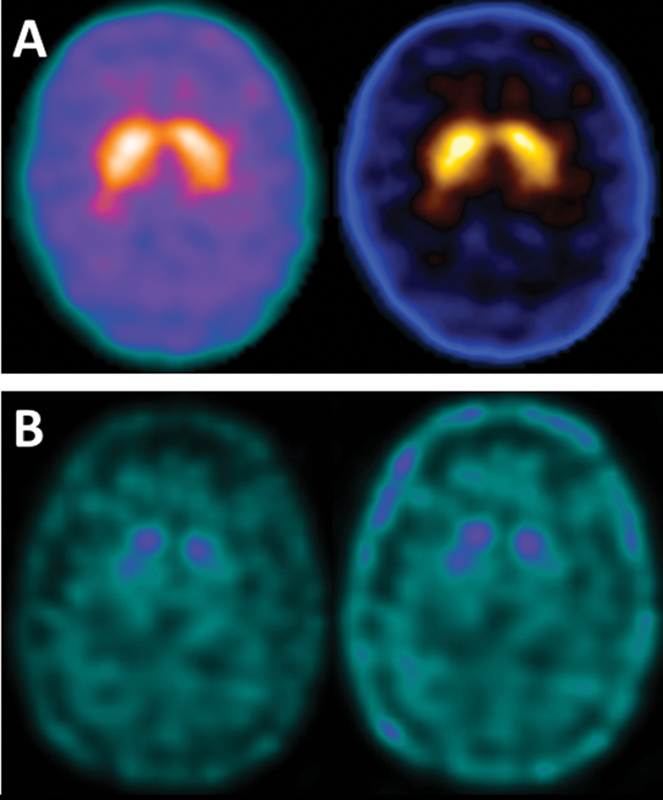
DAT SPECT with ^99m^ Tc-TRODAT-1. ( **A** ) shows a normal DAT SPECT from a healthy subject, while figure ( **B** ) discloses a marked decrease in dopamine in a patient with Parkinson's disease. This image is from the personal archive of the authors.

In the realm of functional neuroimaging, different radiotracers and imaging techniques can access the dopaminergic pathway. Dopamine transporter (DAT) SPECT has largely been used as a reliable test to demonstrate *in vivo* dopaminergic dysfunction, by using ^99m^ Tc-TRODAT-1 (SPECT-TRODAT) transporter, a tracer that is reasonably costly and available. As the name implies, this technique traces presynaptic ligands and its measurement is a valuable imaging method to differentiate PD from its mimics like ET, dystonic tremor, or functional parkinsonism. 57 58 However, DAT SPECT is *not* a reliable test to differentiate PD from atypical parkinsonism, since these conditions usually present with pre-synaptic dopaminergic dysfunction. 59 Attempted to use DAT SPECT to distinguish PD from atypical parkinsonism using measurements of tracers at the putamen and caudate are inconclusive so far. 57 58 SPECT-TRODAT has a higher sensitivity and specificity for measuring the decrement of DAT in PD patients when compared with other imaging techniques. Figure 1A shows a normal DAT SPECT from a healthy subject, while Figure 1B discloses a marked decrease in dopamine receptor binding in a patient with PD.

**Figure 2:**
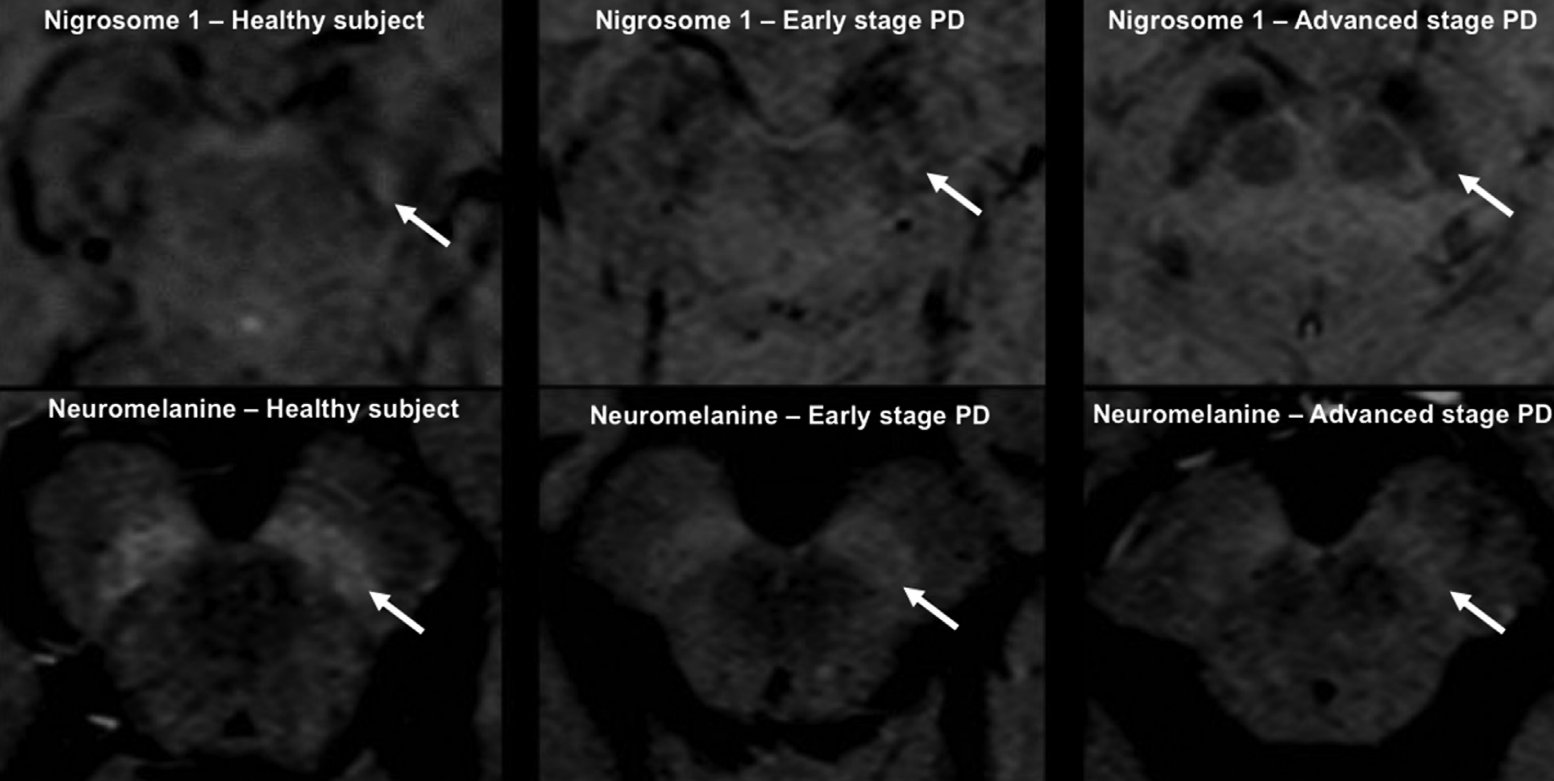
Brain MRI with nigrosome and neuromelanin findings, respectively in healthy subjects, early stage of PD and advanced stage of PD. In healthy subjects there is a clear swallow tail appearance in nigrosome imaging and hyperintense signal in the substantia nigra in the neuromelanine sensitive MRI. On the other hand, in advanced stages of PD, there is absence of the swallow tail appearance in nigrosome imaging and decrease of the hyperintensity in neuromelanine imaging. Early stage of PD presents with intermediate findings between both conditions described above. This image was kindly supplied by Dr. Victor Hugo Rocha Marussi, from Beneficência Portuguesa, São Paulo, Brazil.

Recent techniques of brain MRI to evaluate the substantia nigra in PD have been developed, such as nigrosome and neuromelanin studies, quantitative susceptibility mapping (QSM), and visual assessment of dorsal nigral hyperintensity. 60 Nigrosome 1 is a region of the substantia nigra which is more densely affected in PD. The neuromelanin protocol is performed by using a T1-weighted fast spin echo sequence, while nigrosome is evaluated by T2 sequences. 56 60 Furthermore, nigrosome and neuromelanine evaluation may work as an in vivo marker for the progression of nigral degeneration from early to advanced stages of PD. Finally, neuromelanin-sensitive MRI may differentiate ET from PD, although sensibility and specificity are lower than the DAT SPECT. 60 Figure 2 shows nigrosome and neuromelanin findings in healthy subjects, early stage of PD, and advanced stage of PD.

Positron emission tomography (PET) is a relatively expensive and not widely available technique, which, however, offers high sensitivity with better spatial and temporal resolution compared to other techniques. PET can assess both presynaptic [measurent of aromatic amino acid decarboxylase (AADC) activity ( ^18^ F-DOPA), DAT activity and vesicular monoamine transporter (VMAT2) density (DTBZ)] and post-synaptic activities (i.e., ^11^ C-raclopride binding to striatal D _2_ receptors). As such, these techniques may be useful to facilitate the differential diagnosis of PD when a mixed pre and post-synaptic degenerative form is suspected. 56 61

The substantia nigra can also be evaluated using transcranial sonography. Around 90% of PD patients present with increased echogenicity of the substantia nigra while approximately 10% of healthy subjects and 16% of ET patients also have this finding. 62 Therefore, although transcranial sonography is a low-cost and noninvasive imaging technique to evaluate the dopaminergic pathway, it has less sensitivity and specificity than DAT SPECT and does not have a reliable accuracy for the diagnosis of PD. 62

It is relevant to bear in mind that imaging studies are not methods to diagnose PD. Imaging methods such as DAT SPECT and MRI with nigrosome 1 are helpful in showing dopaminergic dysfunction or parkinsonism. In the absence of parkinsonism, abnormal nigrosome 1 or DAT SPECT does not mean that the individual has or will develop a degenerative parkinsonism.

### Genetic test for Parkinson's disease

The understanding of the etiology and molecular mechanisms of PD had a tremendous progress during the last two decades, especially due to the development of new genomic tests and genetic discoveries. The identification of mutations in genes such as *SNCA* (α-synuclein), *LRRK2* (leucine-rich repeat kinase-2), or *GBA1* (glucocerebrosidase) has allowed a better understanding of the molecular and pathophysiological mechanisms of the hereditary forms and of PD in general. 63 However, although there are currently 25 genetically linked subtypes of PD, genetic testing in clinical practice (single genetic testing or Sanger; genetic panel; or exome sequencing) should only recommended for a minority of patients presenting the following features:

- early onset PD (< 40-year-old);
- consistent family history;
- syndromic forms of parkinsonism with very early onset. 64

In patients with a family history indicating autosomal dominant PD, the *LRRK2* gene should be investigated, especially in the Ashkenazi population. On the other hand, Brazilian patients with early onset or juvenile PD and suspected autosomal recessive disease, PARK2, or *PRKN* gene should be initially tested. 64 65 66

### Cerebrospinal fluid (CSF)

A few potential cerebrospinal fluid (CSF) biomarkers have been investigated in patients with PD, including total α-synuclein, oligomeric α-synuclein, lysosomal enzyme activities, and neurofilament light chain. 67 However, differently from similar techniques used in Alzheimer's disease, PD CSF biomarkers for PD are not currently measured in routine clinical practice, been restricted to research protocols, for example, to investigate and determine pre-symptomatic stages in predisposed subjects. 67

### Other ancillary tests

Other complementary tests could be used in the diagnostic workup of patients with suspected PD, especially when atypical forms of parkinsonism were not ruled out. For instance: cardiac scintigraphy is normal in MSA, and has decreased binding in PD and Lewy body dementia; autonomic tests may be abnormal in early changes in MSA and late changes of PD; polysomnography may disclose RBD in alpha-synucleinopathies. 6 25

In conclusion, the correct diagnosis of PD in the earlier stages and often during the course of the disease is a challenging process. Although treatment at the moment is mainly symptomatic and not disease-modifying from a pathological standpoint, accurate diagnosis remains a pivotal aspect of health care, given its implications regarding adequate approaches to therapeutic interventions and counseling regarding prognosis. This has been an ongoing concern since PD's early descriptions and several endeavors were historically fruitful in advancing the field, leading to the current position where clinicians are well-equipped with knowledge and ancillary resources that have dramatically improved specificity and sensitivity for the diagnosis of PD and its main differential diagnoses. Finally, it is foreseeable that additional layers of challenges and complexity will soon be triggered by the use of artificial intelligence and machine learning models in the context of the diagnosis, prediction, treatment, and prognosis of PD.
